# Combined Brain‐Cardiac CT in Ischemic Stroke: Unveiling Atrial Markers Linked to Large Vessel Occlusion in AF Patients

**DOI:** 10.1111/ene.70079

**Published:** 2025-07-07

**Authors:** Soundous M'Rabet, Gauthier Duloquin, Pierre‐Antoine Garbuio, Angélique Bernard, Thibault Leclercq, Camil‐Cassien Bamdé, Pierre‐Olivier Comby, Frédéric Ricolfi, Yannick Béjot, Charles Guenancia

**Affiliations:** ^1^ Cardiology Department University Hospital Dijon France; ^2^ PEC 2 EA 7460, UFR Sciences de Santé Université de Bourgogne Dijon France; ^3^ Neurology Department University Hospital Dijon France; ^4^ Radiology Department University Hospital Dijon France; ^5^ Vascular Surgery Department University Hospital Dijon France

**Keywords:** atrial cardiomyopathy, atrial fibrillation, CT scan, large vessel occlusion, stroke

## Abstract

**Background:**

Atrial fibrillation (AF) increases the risk and severity of ischemic stroke, particularly in large vessel occlusion (LVO) cases. Atrial cardiomyopathy (AtCM) severity may explain why some AF patients suffer LVO while others develop distal cardioembolic strokes. This study aimed to investigate whether AtCM markers detected on acute brain‐cardiac CT are associated with LVO in AF patients presenting with ischemic stroke.

**Methods:**

We analyzed 619 ischemic stroke patients admitted to Dijon University Hospital from November 2018 to March 2021. The cohort was divided based on the presence of LVO. Cardiac CT assessed left atrium volume (LAV), left atrial thrombus, and epicardial adipose tissue. Patients were grouped by LVO status.

**Results:**

Of the 248 patients with acute stroke and AF, 138 (56%) had LVO. LVO patients had higher NIHSS scores (11 vs. 3, *p* < 0.001) and more frequent use of IV thrombolysis (27.7% vs. 12.3%, *p* = 0.030). Cardiac CT showed higher LAV in LVO patients (112.0 ± 43.0 cm^3^ vs. 100.1 ± 39.0 cm^3^, *p* = 0.026). Multivariable analysis identified female sex (OR 1.98, 95% CI 1.12–3.50, *p* = 0.018) and LAV > 90 mL (OR 2.02, 95% CI 1.18–3.46, *p* = 0.010) as independent predictors of LVO.

**Conclusions:**

Larger LAV and female sex independently predict LVO in AF patients, highlighting AtCM's role in stroke pathophysiology. Further research is needed to explore prevention strategies for high‐risk AF patients.

## Graphical abstract

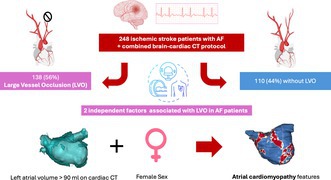

## 1 Introduction

Atrial fibrillation (AF) is a leading cause of ischemic stroke, increasing stroke risk, severity, and mortality [1]. Large vessel occlusion (LVO) accounts for 20%–25% of acute ischemic strokes, primarily in the anterior circulation, with a 4.5‐time higher mortality rate than other AIS cases [2, 3]. AF is more common in LVO strokes, with a reported prevalence of up to 45% [4]. By 2050, ischemic stroke with LVO in the anterior circulation is expected to rise by 51%–81% in France [5], emphasizing the need to understand patients at risk for cardioembolic LVO. Although progress has been made in understanding the pathophysiology of thromboembolism in AF, predicting cardioembolic stroke remains challenging [6, 7].

Recent evidence highlights the concept of atrial cardiomyopathy (AtCM), where the atrium is the primary site of disease. This involves a complex interplay of metabolic, fibrotic, and inflammatory mechanisms [8], manifesting as electrical abnormalities (premature atrial contractions, AF), mechanical disorders (left atrial (LA) stiffness, heart failure with preserved ejection fraction), or thrombo‐embolic events [9]. Cardiac computed tomography (CT) is a valuable tool for assessing AtCM features, such as left atrial volume (LAV) [10], epicardial adipose tissue (EAT) volume and attenuation, as well as the presence of left atrial appendage thrombus (LAAt) [11, 12].

This study investigates whether AtCM parameters, assessed via combined brain‐cardiac CT during acute stroke, are associated with proximal occlusions in patients with AF, aiming to identify predictors of severe cardioembolic stroke.

## 2 Methods

### 2.1 Study Population

We retrospectively studied a cohort of 619 patients with imaging‐confirmed acute ischemic stroke who were consecutively referred to the Dijon University Hospital in France between November 2018 and March 2021.

Patients were excluded if they were younger than 18 years, if contrast‐enhanced CT could not be performed (pregnancy, renal disease, or allergy to iodinated contrast). Only patients with a diagnosis of AF, whether in the history, during hospitalization, or at follow‐up, were included.

The study was conducted in accordance with the Declaration of Helsinki. Institutional policy did not require Institutional Review Board approval.

### 2.2 Imaging Modalities

All patients with a suspicion of ischemic stroke underwent an emergency combined brain and cardiac CT, according to the Dijon University Hospital protocol (Aquilion ONE GENESIS, Canon Medical Systems, Otawara, Japan) [11].

### 2.3 Image Analysis

Images were analyzed by two radiologists using a workstation (Centricity Universal Viewer, GE Healthcare, General Electric Company, United States, version 6.0).

Magnetic resonance imaging (MRI) was performed if a normal brain CT could not solely exclude the stroke diagnosis. Acute stroke was defined on CT by low density corresponding to a vascular territory or prolonged time to peak on the perfusion CT, and on MRI by intense high signal on diffusion‐weighted imaging. The diagnosis was made by a neurologist based on the imaging results and the hospital discharge summary.

In the anterior circulation, LVO was defined as occlusion of the terminal intracranial internal carotid artery, M1 and M2 segments of the middle cerebral artery (including tandem occlusions), or A1 and A2 segments of the anterior cerebral artery. In the posterior circulation, LVO was defined as occlusion of the basilar artery or P1 segment of the posterior cerebral artery [13].

A radiologist with expertise in cardiac and neurological imaging was consulted to review the LAA, and any discrepancies were resolved through consensus.

The volume and attenuation of EAT were determined by manual contouring of the pericardium on axial sections to create a volume. Based on the results of previous studies, a window between −200 and −50HU was defined for the purpose of isolating fat tissue [10, 14]. The software automatically calculated the volume and attenuation of the EAT.

The LAV and LA surface were measured using the same contouring method as previously described [10].

### 2.4 Clinical Data

Medical records were reviewed to determine baseline clinical characteristics and medication status at the time of discharge.

Stroke severity was evaluated using the National Institutes of Health Stroke Scale (NIHSS) score.

### 2.5 Follow‐Up

Patients were scheduled for outpatient visits with a neurologist at 6 months and then annually until further notice, at which point additional visits would be scheduled as needed.

Patients who received an implantable cardiac monitor (ICM) were scheduled for a follow‐up visit with a cardiologist at 6 weeks and then at 3‐month intervals or via remote monitoring for a minimum of 2 years.

### 2.6 Statistical Analysis

Statistical results for categorical variables are presented as percentages (%), for continuous variables as means ± standard deviation for normal distribution, and as medians (first quartile—third quartile) for non‐normal distribution.

The chi‐squared or Fisher's exact test was employed for categorical data, whereas the Student's *t*‐test was used to assess continuous data with normal distribution variables, and the Mann–Whitney test was applied for non‐parametric variables. The optimal threshold to discriminate LVO using LAV was obtained with the receiver‐operating characteristic (ROC) curve with the best sensitivity and specificity according to the Youden index. The multivariable model included variables according to their univariable relationship with LVO (*p* < 0.20), and analyses were performed using logistic regression model to identify factors associated with LVO, with results expressed as odds ratios (OR) (95% confidence interval). A *p*‐value of < 0.05 was considered statistically significant. Analyses were performed with SPSS software (26.0, IBM Inc., USA).

## 3 Results

### 3.1 Patients' Characteristics

**FIGURE 1:**
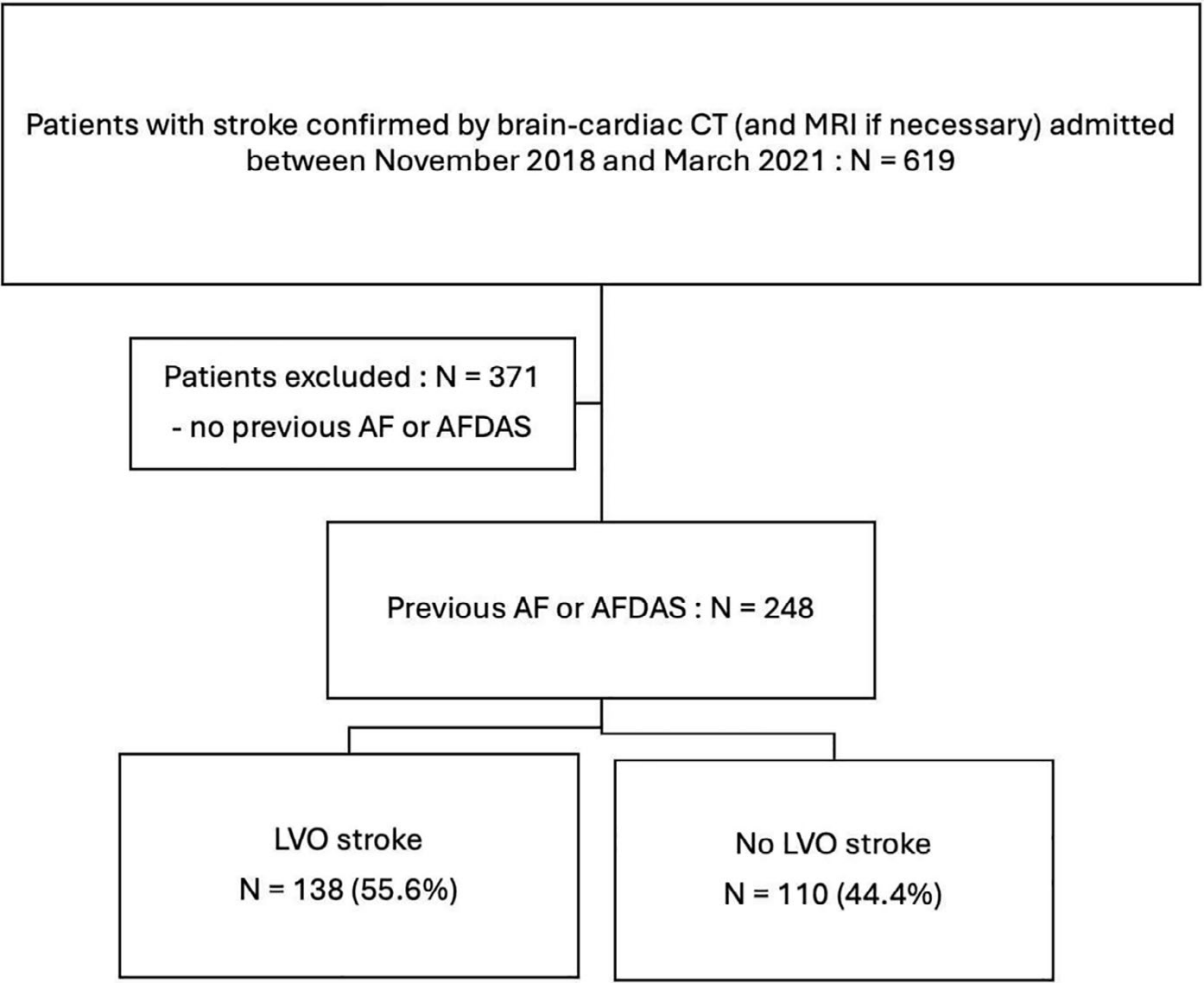
Flowchart. AF, Atrial fibrillation; AFDAS, Atrial fibrillation diagnosed after stroke; CT, Computed tomography; LVO, Large vessel occlusion; MRI, magnetic resonance imaging.

Among 619 patients who were diagnosed with a confirmed stroke during the study period, 248 (40%) had AF, either known before admission, diagnosed during hospitalization or at follow‐up. The study population was divided into two groups based on the presence or absence of LVO on the brain CT scan, as shown in Figure 1.

Patient characteristics are shown in Table 1. Compared to patients without LVO, those with LVO were more frequently women (63.0% vs. 47.3%, *p* = 0.013). In more than half of the cases, AF was already known (56.4% vs. 51.4%, respectively, in the non‐LVO and LVO group, *p* = 0.441). A history of ischemic stroke was present in nearly a quarter of the patients, with no significant difference between the two groups. The CHA_2_DS_2_‐VASc score did not differ between the two groups.

**TABLE 1:** Patients' clinical and biological characteristics.

	No LVO *N* = 110	LVO *N* = 138	*p*
**Demographic characteristics**			
Age (years)	84 (76–89)	82 (72–88)	0.583
Female sex	52 (47.3%)	87 (63.0%)	**0.013**
**Cardiovascular risk factors**			
BMI (kg/m^2^)	25.8 ± 5.8	26.4 ± 6.8	0.568
Hypertension	91 (82.7%)	114 (82.6%)	0.980
Diabetes	30 (27.3%)	30 (21.7%)	0.312
Current smoking	15 (13.6%)	16 (11.6%)	0.629
Dyslipidemia	54 (49.1%)	69 (50.0%)	0.887
**Other comorbidities**			
Obstructive sleep apnea syndrome	12 (10.9%)	17 (12.3%)	0.731
Peripheral artery disease	9 (8.2%)	9 (6.5%)	0.631
Coronary artery disease	22 (20.0%)	26 (18.8%)	0.818
Dilated cardiomyopathy	7 (6.4%)	11 (8.0%)	0.806
Previous ischemic stroke	29 (26.4%)	28 (20.3%)	0.259
Valvular surgery	11 (10.0%)	8 (5.8%)	0.237
**Diagnosis of AF**			
Previous AF	62 (56.4%)	71 (51.4%)	0.441
AF diagnosed during hospitalization	36 (32.7%)	50 (36.2%)	0.517
AF diagnosed at follow‐up	12 (10.9%)	17 (12.3%)	0.854
ICM implantation	6 (13.3%)	8 (11.9%)	0.909
**CHA**_**2**_ **DS**_**2**_ **‐VASc score**	4 (3–5)	4 (3–5)	0.997
*Biological data at admission*			
Hemoglobin (g/dL)	13.7 ± 7.9	12.9 ± 1.9	0.242
NT‐pro BNP (ng/L)	3785 ± 7195	2768 ± 4587	0.390
Troponin (ng/L)	0.8 ± 2.8	1.1 ± 5.7	0.630
CRP (mg/L)	63.9 ± 320.0	19.0 ± 39.4	0.120
Mean platelet volume (fL)	10.7 ± 1.0	11 ± 3.4	0.392
TSH (mUI/L)	2.0 ± 2.6	1.6 ± 1.2	0.189

*Note:* *n* (%), mean (± SD), median (25th—75th percentile). Bold indicates *p* < 0.05.

Abbreviations: AF, atrial fibrillation; BMI, body mass index; CHA_2_DS_2_‐VASc, congestive heart failure, hypertension, age ≥ 75 years (2 points), diabetes mellitus, prior stroke/TIA/thromboembolism (2 points), vascular disease, age 65–74 years, female sex; CRP, C‐reactive protein; ICM, Implantable cardiac monitor; LVO, large vessel occlusion; NT‐pro BNP, N‐terminal pro b‐type natriuretic peptide; TSH, thyroid‐stimulating hormone.

The distribution of cardiovascular risk factors was found to be similar between the two groups. The groups did not differ in terms of biological parameters at admission such as hemoglobinemia, mean platelet volume, troponin, thyroid‐stimulating hormone, N‐terminal pro b‐type natriuretic peptide, and C‐reactive protein levels.

### 3.2 Stroke Severity and Management

Patients with LVO experienced more severe symptoms at both admission and discharge, as indicated by greater NIHSS scores (Table 2). The mean NIHSS score at initial presentation was 11 [5, 6, 7, 8, 9, 10, 11, 12, 13, 14, 15, 16, 17, 18, 19, 20] in the LVO group and 3 [1, 2, 3, 4, 5, 6, 7, 8] in the non‐LVO group (*p* < 0.001). At discharge, the mean NIHSS score had improved to 2 (0–5) and 1 (0–3), respectively (*p* < 0.001).

**TABLE 2:** Stroke severity, management and imaging characteristics.

	No LVO *N* = 110	LVO *N* = 138	*p*
***Stroke severity and management***			
NIHSS score at admission	3 (1–8)	11 (5–20)	**< 0.001**
NIHSS score at discharge	1 (0–3)	2 (0–5)	**< 0.001**
Bleeding complication	2 (2.6%)	7 (7.4%)	0.190
Thrombolysis	13 (12.3%)	38 (27.7%)	**0.030**
Thrombectomy	0	51 (37.2%)	**< 0.001**
1‐year mortality	33 (33.7%)	40 (33.3%)	0.958
***Discharge medical treatment***			
Aspirin	15 (13.6%)	20 (14.5%)	0.847
Aspirin + clopidogrel	3 (0.9%)	6 (4.3%)	0.498
Direct oral anticoagulant	55 (50.0%)	58 (42.0%)	0.210
Vitamin K antagonist	8 (7.3%)	14 (10.1%)	0.429
Direct oral anticoagulant + aspirin	9 (8.2%)	6 (4.3%)	0.208
**Cerebral computed tomography data**			
Multiple location of ischemic lesion	18 (16.4%)	5 (3.6%)	**0.001**
**Cardiac computed tomography data**			
LAA thrombus	20 (18.2%)	35 (25.4%)	0.176
LV thrombus	1 (0.9%)	1 (0.7%)	1
LA surface (cm^2^)	26.1 ± 7.1	29.2 ± 9.5	**0.005**
LA volume (cm^3^)	100.1 ± 39.0	112.0 ± 43.0	**0.026**
Mean EAT attenuation (HU)	−86.4 ± 5.3	−85.5 ± 4.3	0.134
EAT volume (cm^3^)	88.3 ± 54.0	78.0 ± 47.0	0.132
Coronary plaques	93 (84.5%)	104 (75.4%)	0.075
**LAA shape**			
Windsock	71 (64.5%)	87 (63.0%)	0.806
Chicken	19 (17.3%)	26 (18.8%)	0.750
Cactus and cauliflower	20 (18.2%)	25 (18.1%)	1
***Echocardiography data***			
TTE	55 (50.5%)	67 (48.2%)	0.798
LA surface (cm^2^)	27.4 ± 11.6	26.2 ± 6.0	0.614
LA volume (mL/m^2^)	42.2 ± 23.0	46.5 ± 17.3	0.453
LVEF (%)	53.5 ± 12.3	55.3 ± 12.1	0.468
TEE	3 (2.8%)	8 (5.8%)	0.356

*Note:* *n* (%), mean (± SD), median (25th–75th percentile). Bold indicates *p* < 0.05.

Abbreviations: EAT, epicardial adipose tissue; HU, Hounsfield Unity; LA, left atrium; LAA, left atrial appendage; LV, left ventricle; LVEF, left ventricle ejection fraction; LVO, large vessel occlusion; NIHSS, national institutes of health stroke scale; TEE, transesophageal echocardiography; TTE, transthoracic echocardiography.

Regarding acute stroke management, mechanical thrombectomy was exclusively employed in cases of large vessel occlusion (37.2% vs. 0%, *p* < 0.001), and intravenous thrombolysis was more commonly administered in the LVO group compared to the non‐LVO group (27.7% vs. 12.3%, *p* = 0.03).

Anticoagulant therapy was the predominant approach to medical discharge in both groups, with 65.4% and 56.6% of non‐LVO and LVO stroke patients, respectively, receiving either a vitamin K antagonist or a direct oral anticoagulant at hospital discharge. A total of 16.4% of patients in the non‐LVO group and 18.8% of patients in the LVO group were discharged on antiplatelet therapy alone, mainly because they were classified as cryptogenic at discharge and were diagnosed with AF during follow‐up.

At 1 year, there was no significant difference in case‐fatality rates between the two groups (33.7% vs. 33.3%, *p* = 0.958).

### 3.3 Imaging Characteristics

As shown in Table 2, LVO strokes were more likely to affect a unique vascular territory (3.6% vs. 16.4%, *p* = 0.001). The presence of LAAt or left ventricular (LV) thrombus was not a predictive factor for LVO stroke (18.2% vs. 25.4%, *p* = 0.176; and 0.9% vs. 0.7%, *p* = 1, respectively).

LA dimensions observed on cardiac CT were found to be significantly higher in the presence of LVO, with mean values of 29.2 ± 9.5 cm^2^ for LA surface and 112.0 ± 43.0 cm^3^ for LAV versus 26.1 ± 7.1 cm^2^ (*p* = 0.005) and 100.1 ± 39.0 cm^3^ (*p* = 0.026), respectively, in the absence of LVO.

These findings were not replicated in the TTE analysis of LA dimensions.

EAT volume and mean EAT attenuation were not significantly different between the two groups. There was no significant difference in the presence of coronary plaques, LAA shape, or LV ejection fraction estimated by TTE.

After ROC curve analysis, the LAV value that was best able to predict LVO stroke on CT was > 90 mL, with a sensitivity of 65% and a specificity of 52% (area under the curve: 0.591; 95% CI: 0.52–0.66 *p* = 0.013).

### 3.4 Factors Associated With LVO Stroke

In multivariable analysis (Table 3), only two factors were independently associated with LVO in patients with AF: LA dilatation as assessed according to the previously described threshold of LAV > 90 mL on CT (OR 2.02; 95% CI 1.18–3.46; *p* = 0.010) and female sex (OR 1.98; 95% CI 1.12–3.50; *p* = 0.018).

**TABLE 3:** Multivariable logistic regression analysis of LVO‐stroke associated factors in patients with AF.

	Univariable			Multivariable		
	OR	95% CI	*p*	OR	95% CI	*p*
Female sex	1.90	1.14–3.17	0.013	1.98	1.12–3.50	**0.018**
LAA thrombus	1.53	0.82–2.84	0.178	1.09	0.55–2.16	0.814
Coronary artery disease	0.51	0.27–1.00	0.051	0.51	0.26–1.01	0.052
LAV > 90 mL	2.08	1.25–3.48	0.005	2.02	1.18–3.46	**0.010**
EAT volume	1.00	0.99–1. 00	0.133	1.00	0.99–1.01	0.772
Mean EAT attenuation	1.04	0.99–1.10	0.136	1.03	0.97–1.09	0.348
Age	0.99	0.97–1.02	0.581			
TSH	0.89	0.76–1.05	0.175			
Previous ischemic stroke	0.71	0.39–0.71	0.260			
Anticoagulant therapy	0.74	0.44–1.26	0.273			
Hypertension	0.99	0.51–1.92	0.980			
Diabetes	0.74	0.41–1.33	0.313			
**LAA shape**						
Windsock	ref	ref	0.95			
Chicken	1.11	0.57–2.18	0.75			
Cactus and cauliflower	1.02	0.52–1.99	0.95			

*Note:* Bold indicates *p* < 0.05.

Abbreviations: AF, atrial fibrillation; CI, confidence interval; CT, computed tomography; EAT, epicardial adipose tissue; HR, hazard ratio; LAA, left atrial appendage; LAV, left atrial volume; LVO, large vessel occlusion; OR, odds ratio; ref, reference; TSH, thyroid‐stimulating hormone.

## 4 Discussion

The present study suggests that two factors may be independently associated with LVO in ischemic stroke patients and AF: female sex and LA dilatation with a 90‐mL threshold on cardiac CT.

### 4.1 Women and Large Vessel Occlusion

The observed association between the female sex and the presence of large vessel occlusion could be attributed to the inherent higher cardioembolic risk in women.

The effect of female sex on thromboembolic events in atrial fibrillation remains controversial and is now considered a stroke risk modifier rather than a risk factor per se [15]. As a result of this controversy, the CHA_2_DS_2_‐VA score, excluding the sex category, is now the predominant approach for estimating thromboembolic risk in AF and initiating anticoagulation therapy, according to the latest ESC guidelines for the management of AF [16]. The median CHA_2_DS_2_‐VASc scores of LVO patients were comparable to those of non‐LVO patients, suggesting that this score does not adequately predict the risk of large vessel occlusion in patients with atrial fibrillation. These results raise the question of the performance of this score in predicting cardioembolic stroke rather than all‐causes ischemic stroke, particularly within a context where its predictive performance has already been subject to question [17].

Several hypotheses have been proposed to explain the mechanisms by which the female sex modifies stroke risk. The female sex is in itself a major contributor to AtCM as demonstrated by Winters et al., with an effect size on AtCM comparable to that of persistent AF or heart failure [18]. Women with AF have a greater burden of atrial fibrosis as evidenced by two studies that employed atrial delayed enhancement assessment via cardiac MRI [19, 20]. Left atrial fibrosis, underlying atrial dysfunction in cardiomyopathy [21], is independently associated with stroke risk [22]. Women may have more fibrosis due a subclinical proinflammatory state, attributed to higher levels of C‐reactive protein and fibroblast growth factor‐23 [23], particularly associated with cardioembolic stroke but not other types [24]. Reduced levels of nitric oxide (NO) caused by downregulation of the NO synthase in women also contribute to the thrombogenic endocardial remodeling in AF [25]. Inflammation may drive AF onset, maintenance and thrombogenicity [26, 27].

Second, despite improved physician awareness, women are less likely than men to receive appropriate anticoagulant therapy [28], statins or antiplatelet agents [29] leading to worse comorbidity control exacerbating atrial dysfunction. Comparison of medical treatment at the time of admission and pre‐stroke cardiovascular risk management would have been insightful. Some patients did not receive anticoagulants at discharge due to delayed AF diagnosis, mostly on ICM, or delayed initiation because of infarct size.

Finally, another hypothesis to explain our findings is that women are more susceptible to LVO due to a smaller vessel diameter compared to men for the same thrombus size. Carotid artery diameter is smaller in women, even after adjusting for body and neck size, age, and blood pressure [30]. Van Der Meij et al. found increased intracranial vessel tortuosity in women in a population of patients with LVO stroke treated by endovascular thrombectomy [31]. Moreover, intracerebral arterial diameters were found to be significantly reduced in female subjects compared to their male counterparts [32]. Measurement and characterization of supra‐aortic vessels on a brain‐cardiac CT scan may provide additional information to support this hypothesis.

### 4.2 Left Atrial Dilatation and Large Vessel Occlusion

Left atrial dilatation is a major component of AtCM and is associated with an increased risk of ischemic stroke [33, 34], with a worse initial stroke severity [35]. In a study design like ours, Butt et al. demonstrated that patients with LVO stroke had a larger LA diameter measured on non‐ECG gated cardiac CT in the acute phase compared to patients with non‐LVO stroke [36]. There was no association between atrial fibrillation and LVO, likely due to the lack of intensive screening for atrial fibrillation, lack of follow‐up, and relatively small sample size.

The present study proposes a 90‐mL threshold on cardiac CT scans as the optimal value for predicting LVO in stroke patients with AF. This threshold is consistent with our previous findings, in which we identified a threshold of 85 mL on cardiac CT scans to best predict AF diagnosed after stroke in patients presenting without previous AF [10]. In the study conducted by Shin et al., left atrial enlargement with LAV indexed > 27 mL/m^2^ on TTE was associated with cardioembolic stroke with an adjusted OR of 6.7 [37], irrespective of the AF status. In Yaghi et al.'s study, moderate to severe left atrial dilatation (anteroposterior diameter > 43 mm in women, > 47 mm in men) on TTE was associated with the recurrence of cryptogenic and cardioembolic stroke, even after adjusting for AF, with a 2.4‐fold risk increase [38].

Studies suggest LA dilatation may indicate undiagnosed paroxysmal AF, increasing stroke risk [39, 40]. To our knowledge, our study is the first to suggest that patients with LA dilatation may be at increased risk of more proximal cardioembolic strokes compared with patients with normal‐sized LA in patients already diagnosed with AF. The mechanisms are unclear, but it is hypothesized that an enlarged LA promotes hemostasis, with spontaneous echocardiographic contrast or LAAt more common in these patients [41].

Our findings supports the concept of thrombogenic endocardial remodeling in AtCM, as described in the EHRA/HRS/APHRS/LAHRS 2024 consensus [9]. It has been suggested that fibrotic remodeling of the atrial wall may lead to thrombus formation independent of the presence of AF. Furthermore, immunohistochemical analysis of excised LAAs revealed a significant association between endocardial endothelial damage and LAAt, as well as ischemic stroke history, independent of AF [42]. The association between AtCM and stroke is therefore direct, bypassing AF and primarily attributable to the pro‐thrombotic nature of the diseased atrium.

The results differed between TTE and cardiac CT due to TTE's limitations in measuring volume and surface accurately, as well as potential inter‐operator variability. This may illustrate one of the constraints of the ARCADIA study, which found no benefit apixaban over aspirin in preventing cryptogenic stroke recurrence in patients exhibiting AtCM markers, including P‐wave terminal force in ECG lead V1, serum NT‐proBNP, and LA diameter, which was measured by echocardiogram [43]. The results of this study, in conjunction with those of our previous investigation, suggest that the assessment of AtCM parameters on CT scans, when performed according to a brain‐cardiac CT scan protocol, may represent a superior approach in identifying the patients at elevated embolic risk [10].

### 4.3 Age and Large Vessel Occlusion

Despite its established role in assessing cardioembolic risk in patients with AF, age did not prove to be a significant predictor of LVO in the study population. These findings align with those reported by Winters et al., who observed a minimal association between age and the extent of atrial cardiomyopathy, as indicated by atrial fibrosis, on histological examination of LAA, when compared to the impact of persistent AF or heart failure [18].

It is also possible that the presence of LVO does not reflect a group of AF patients at higher thromboembolic risk, but rather a larger thrombus size resulting in more proximal vessel occlusion. The mechanisms underlying the determination of thrombus size remain to be elucidated.

### 4.4 Limitations

The limitations of this study include its single‐center and retrospective design, which may introduce a selection bias. The EAT volume was measured throughout the heart, whereas these measurements are known to be associated with AtCM when taken from the left atrium, particularly the posterior wall [44].

Given that the pattern of AF has been shown to have a significant impact on stroke rate [45] and clinical outcome [46], it would be interesting to investigate the interaction between the characteristics (type, burden, clinical presentation) of known AF and the risk of LVO stroke in AF patients. However, there was no association between the temporality of diagnosis of AF (previously known or diagnosed after stroke) and the presence of LVO in our study.

To date, trans‐esophageal echocardiography (TEE) remains the gold standard for the assessment of LAAt. However, the comparison of CT findings to TEE data was limited because it was performed in only 11 (4.4%) patients in the total study population. With the introduction of cardiac CT angiography in our routine acute stroke imaging protocol in 2018 [11], TEE has practically become redundant and has been used primarily to rule out patent foramen ovale. The growing body of research supporting the use of cardiac CT as an alternative mean of assessing LAAt is a prominent factor in this regard, with a sensitivity and sensibility up to 100% in diagnosis LAAt using delayed contrast‐enhanced cardiac CT [47, 48, 49].

Finally, we were unable to measure the diameter of the supra‐aortic vessels on brain‐cardiac CT between men and women to support the hypothesis of a role of sex in the occurrence of LVO.

## 5 Conclusion

In this single‐center retrospective study using combined brain‐cardiac CT in AF patients with ischemic stroke, female sex and LA dilatation were linked to LVO. Our findings support the role of AtCM in AF thrombogenesis. Further studies are needed to assess alternative pharmacological agents and more aggressive prevention strategies for high‐risk AF patients, given the expected economic and social burden of these strokes.

## Author Contributions

**Soundous M'Rabet:** writing – original draft, writing – review and editing. **Gauthier Duloquin:** supervision, methodology, conceptualization. **Pierre‐Antoine Garbuio:** data curation. **Angélique Bernard:** supervision, writing – review and editing, conceptualization, software. **Thibault Leclercq:** supervision, software, conceptualization. **Camil‐Cassien Bamdé:** supervision. **Pierre‐Olivier Comby:** supervision, software, conceptualization. **Frédéric Ricolfi:** supervision, conceptualization, software. **Yannick Béjot:** supervision, writing – review and editing, validation, methodology, conceptualization. **Charles Guenancia:** writing – review and editing, supervision, validation, methodology, conceptualization.

## Conflicts of Interest

The authors declare no conflicts of interest.

## Data Availability

The data that support the findings of this study are available from the corresponding author upon reasonable request.
